# 6-Methyl-*N*-(4-methoxy­phen­yl)-2-[(*E*)-(4-methyl­phen­yl)methyl­eneamino]-4,5,6,7-tetra­hydro­thieno[2,3-*c*]pyridine-3-carboxamide

**DOI:** 10.1107/S1600536808017236

**Published:** 2008-06-13

**Authors:** G. N. Anilkumar, M. K. Kokila, Puttaraja, S. Mohan, J. Saravanan

**Affiliations:** a Department of Physics, MS Ramaiah Institute of Technology, MSRIT Post, Bangalore 560 054, Karnataka, India; b Department of Physics, Bangalore University, Bangalore 560 056, Karnataka, India; c PES College of Pharmacy, Hanumanthanagar, Bangalore 560 050, Karnataka, India

## Abstract

The molecular structure of the title compound, C_24_H_25_N_3_O_2_S, is stabilized by intra­molecular N—H⋯N, C—H⋯O and C—H⋯S hydrogen bonds. There are no significant inter­molecular inter­actions.

## Related literature

For related literature, see: Gewald *et al.* (1966 ▶); Cohen *et al.* (1977 ▶); Csaszar & Morvay (1983 ▶); Lakshmi *et al.* (1985 ▶); Mohan & Saravanan (2003 ▶); Dzhurayev *et al.* (1992 ▶); Sebnis *et al.* (1999 ▶); Anilkumar *et al.* (2005 ▶); El-Maghraby, Haroun & Mohamed (1984 ▶).
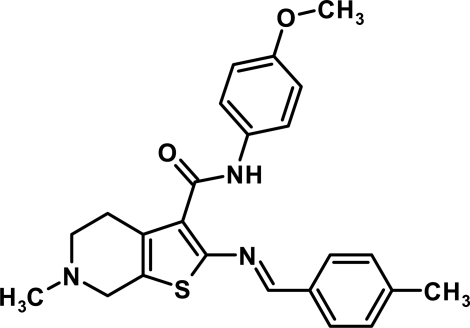


## Experimental

### 

#### Crystal data

C_24_H_25_N_3_O_2_S
*M* *_r_* = 419.53
Triclinic, 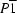

*a* = 8.3905 (11) Å
*b* = 9.9883 (13) Å
*c* = 12.9549 (17) Å
α = 91.375 (2)°
β = 94.789 (3)°
γ = 96.121 (2)°
*V* = 1075.2 (2) Å^3^
*Z* = 2
Mo *K*α radiation
μ = 0.18 mm^−1^
*T* = 292 (2) K
0.32 × 0.28 × 0.22 mm

#### Data collection

Bruker SMART CCD area-detector diffractometer
Absorption correction: multi-scan (*SADABS*; Sheldrick, 1996 ▶) *T*_min_ = 0.942, *T*_max_ = 0.967
10653 measured reflections
3967 independent reflections
2633 reflections with *I* > 2σ(*I*)
*R*_int_ = 0.051

#### Refinement

*R*[*F* ^2^ > 2σ(*F* ^2^)] = 0.085
*wR*(*F* ^2^) = 0.183
*S* = 1.14
3967 reflections
274 parameters
H-atom parameters constrained
Δρ_max_ = 0.26 e Å^−3^
Δρ_min_ = −0.24 e Å^−3^

### 

Data collection: *SMART* (Bruker, 1998 ▶); cell refinement: *SMART*; data reduction: *SAINT* (Bruker, 1998 ▶); program(s) used to solve structure: *SHELXS86* (Sheldrick, 2008 ▶); program(s) used to refine structure: *SHELXL97* (Sheldrick, 2008 ▶); molecular graphics: *ORTEP-3 for Windows* (Farrugia, 1997 ▶); software used to prepare material for publication: *PARST* (Nardelli, 1995 ▶) and *PLATON* (Spek, 2003 ▶).

## Supplementary Material

Crystal structure: contains datablocks global, I. DOI: 10.1107/S1600536808017236/hg2409sup1.cif

Structure factors: contains datablocks I. DOI: 10.1107/S1600536808017236/hg2409Isup2.hkl

Additional supplementary materials:  crystallographic information; 3D view; checkCIF report

## Figures and Tables

**Table 1:** Hydrogen-bond geometry (Å, °)

*D*—H⋯*A*	*D*—H	H⋯*A*	*D*⋯*A*	*D*—H⋯*A*
N2—H2⋯N1	0.86	2.12	2.807 (4)	137
C1—H1⋯S1	0.93	2.60	3.051 (4)	110
C16—H16⋯O1	0.93	2.27	2.863 (5)	121

**Fig. 1.**
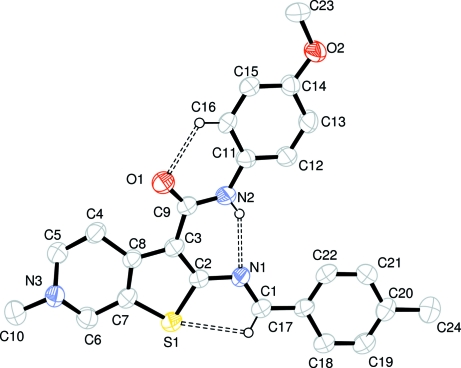
The molecular structure of the title compound, showing the atom-labeling scheme with displacement ellipsiods drawn at the 50% probability level. Dashed lines indicates intramolecular H-bonds.

**Fig. 2.**
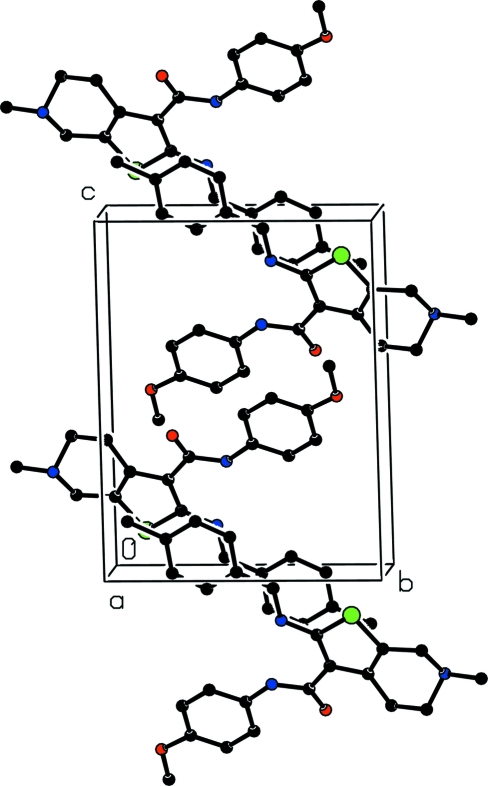
Packing diagram of (I); all H-atoms are omitted for clarity.

## supplementary crystallographic information

### Comment

The title compound (I) was one amongst bicyclic tetrahydropyridinothiophenes (Sebnis *et al.*, 1999), found to exhibit antimicrobial and anti-inflammatory activities. Schiff bases (Csaszar & Morvay, 1983; Lakshmi *et al.*, 1985; Cohen *et al.*, 1977) and their thiophene derivatives (El-Maghraby *et al.*, 1984; Dzhurayev *et al.*, 1992; Gewald *et al.*, 1966) were known for their wide range of biological activities such as antibacterial, antifungal and antitubercular activities. Sulfur containing Schiff bases are particularly effective.(Mohan & Saravanan, 2003).

The bicyclic system exhibits non-planarity, the N—CH_3_ group shows a significant deviation from the molecular plane. The *p*-methoxyphenyl ring (C11–C16/O2/C23) and 4- methylphenyl ring (C17–C22/C24) make dihedral angles of 7.7 (2) and 10.2 (3)°, respectively, with thiophene ring. The torsion angles C3—C9—N2—C11 and C2—N1—C1—C17 show the anti conformation of the two units about the C9—N2 and N1—C1 bonds. In (I) (Fig. 1), intramolecular N2—H2···N1 and C16—H16···O1 hydrogen bonds form pseudo-six-membered rings, while the intramolecular C1—H1···S1 hydrogen bond forms a pseudo-fivemembered ring, thus locking the molecular conformation and eliminating conformational flexibility. (Anilkumar *et al.* 2005). Molecules are arranged in zigzag layers viewed along *a* axis.(Fig 2).

### Experimental

The title compound, (I), was synthesized using the Gewald reaction (Gewald *et al.*, 1966). 4-Methoxyphenyl 2-cyanoacetamide (0.04 mol) was refluxed with *N*-methylpiperidin-4-one (0.04 mol) in the presence of ammonium acetate (1.00 g) and glacial acetic acid (2 ml) in benzene.This mixture was treated with sulfur (1.28 g, 0.04 mol), dimethylamine (4 ml) and ethanol at 323 K. The product was treated with 4- methyl benzaldehyde in an equimolar ratio in the presence of 2- propanol and a catalytic amount of glacial acetic acid under microwave irradiation, which yielded (I). This was purified and crystallized from *N*,*N*-dimethylformamide and ethanol (1:2) by slow evaporation.

### Refinement

H atoms were positioned geometrically, with N—H = 0.86 and C— H = 0.93, 0.97 and 0.96 A° for aromatic, methylene and methyl H, respectively, and constrained to ride on their parent atoms, with *U*_iso_(H) = xUeq(C,*N*), where *x* =1.5 for methyl H and *x* = 1.2 for all other H atoms. A rotating group model was used for methyl groups.

### Crystal data

**Table:**

C_24_H_25_N_3_O_2_S	*Z* = 2
*M**_r_* = 419.53	*F*_000_ = 444
Triclinic, *P*1	*D*_x_ = 1.296 Mg m^−^^3^
Hall symbol: -P 1	Mo *K*α radiation λ = 0.71073 Å
*a* = 8.3905 (11) Å	Cell parameters from 380 reflections
*b* = 9.9883 (13) Å	θ = 1.6–28.6º
*c* = 12.9549 (17) Å	µ = 0.18 mm^−^^1^
α = 91.375 (2)º	*T* = 292 (2) K
β = 94.789 (3)º	Block, yellow
γ = 96.121 (2)º	0.32 × 0.28 × 0.22 mm
*V* = 1075.2 (2) Å^3^	

### Data collection

**Table:**

Bruker SMART CCD area-detector diffractometer	3967 independent reflections
Radiation source: fine-focus sealed tube	2633 reflections with *I* > 2σ(*I*)
Monochromator: graphite	*R*_int_ = 0.051
*T* = 291(2) K	θ_max_ = 25.5º
ψ and ω scans	θ_min_ = 1.6º
Absorption correction: multi-scan(SADABS; Sheldrick, 1996)	*h* = −10→10
*T*_min_ = 0.942, *T*_max_ = 0.967	*k* = −12→12
10653 measured reflections	*l* = −15→15

### Refinement

**Table:**

Refinement on *F*^2^	Secondary atom site location: difference Fourier map
Least-squares matrix: full	Hydrogen site location: inferred from neighbouring sites
*R*[*F*^2^ > 2σ(*F*^2^)] = 0.085	H-atom parameters constrained
*wR*(*F*^2^) = 0.184	*w* = 1/[σ^2^(*F*_o_^2^) + (0.0919*P*)^2^ + 0.1278*P*] where *P* = (*F*_o_^2^ + 2*F*_c_^2^)/3
*S* = 1.14	(Δ/σ)_max_ = 0.001
3967 reflections	Δρ_max_ = 0.26 e Å^−^^3^
274 parameters	Δρ_min_ = −0.24 e Å^−^^3^
Primary atom site location: structure-invariant direct methods	Extinction correction: none

### Special details

**Table:**

Geometry. All e.s.d.'s (except the e.s.d. in the dihedral angle between two l.s. planes) are estimated using the full covariance matrix. The cell e.s.d.'s are taken into account individually in the estimation of e.s.d.'s in distances, angles and torsion angles; correlations between e.s.d.'s in cell parameters are only used when they are defined by crystal symmetry. An approximate (isotropic) treatment of cell e.s.d.'s is used for estimating e.s.d.'s involving l.s. planes.
Refinement. Refinement of *F*^2^ against ALL reflections. The weighted *R*-factor *wR* and goodness of fit *S* are based on *F*^2^, conventional *R*-factors *R* are based on *F*, with *F* set to zero for negative *F*^2^. The threshold expression of *F*^2^ > σ(*F*^2^) is used only for calculating *R*-factors(gt) *etc*. and is not relevant to the choice of reflections for refinement. *R*-factors based on *F*^2^ are statistically about twice as large as those based on *F*, and *R*- factors based on ALL data will be even larger.

### Fractional atomic coordinates and isotropic or equivalent isotropic displacement parameters (Å^2^)

**Table:**

	*x*	*y*	*z*	*U*_iso_*/*U*_eq_	
S1	0.15650 (12)	0.11892 (10)	0.10349 (7)	0.0563 (3)	
O1	0.5787 (3)	0.2412 (3)	0.3856 (2)	0.0713 (9)	
O2	0.9097 (3)	0.8527 (2)	0.5084 (2)	0.0604 (7)	
N1	0.3079 (3)	0.3800 (3)	0.1240 (2)	0.0471 (7)	
N2	0.5036 (4)	0.4298 (3)	0.3103 (2)	0.0523 (8)	
H2	0.4367	0.4575	0.2637	0.063*	
N3	0.2336 (4)	−0.2086 (3)	0.2703 (2)	0.0522 (8)	
C1	0.2503 (5)	0.4032 (4)	0.0322 (3)	0.0551 (10)	
H1	0.1949	0.3317	−0.0078	0.066*	
C2	0.2865 (4)	0.2518 (3)	0.1637 (3)	0.0442 (9)	
C3	0.3661 (4)	0.2116 (3)	0.2540 (3)	0.0433 (9)	
C4	0.3748 (5)	−0.0062 (4)	0.3650 (3)	0.0516 (10)	
H4A	0.4844	−0.0254	0.3582	0.062*	
H4B	0.3741	0.0474	0.4283	0.062*	
C5	0.2676 (5)	−0.1373 (4)	0.3715 (3)	0.0568 (10)	
H5A	0.3195	−0.1948	0.4200	0.068*	
H5B	0.1670	−0.1185	0.3976	0.068*	
C6	0.1331 (5)	−0.1315 (4)	0.2017 (3)	0.0556 (10)	
H6A	0.0266	−0.1339	0.2259	0.067*	
H6B	0.1224	−0.1715	0.1321	0.067*	
C7	0.2076 (4)	0.0119 (4)	0.1999 (3)	0.0469 (9)	
C8	0.3183 (4)	0.0725 (3)	0.2738 (3)	0.0415 (8)	
C9	0.4911 (4)	0.2950 (4)	0.3227 (3)	0.0479 (9)	
C10	0.1536 (5)	−0.3431 (4)	0.2811 (3)	0.0664 (12)	
H10A	0.0545	−0.3371	0.3121	0.100*	
H10B	0.2219	−0.3938	0.3244	0.100*	
H10C	0.1315	−0.3874	0.2141	0.100*	
C11	0.6118 (4)	0.5304 (3)	0.3637 (3)	0.0460 (9)	
C12	0.6143 (5)	0.6609 (4)	0.3305 (3)	0.0596 (11)	
H12	0.5460	0.6787	0.2734	0.072*	
C13	0.7141 (5)	0.7643 (4)	0.3789 (3)	0.0603 (11)	
H13	0.7138	0.8508	0.3538	0.072*	
C14	0.8163 (4)	0.7420 (4)	0.4655 (3)	0.0466 (9)	
C15	0.8154 (5)	0.6133 (4)	0.4991 (3)	0.0573 (10)	
H15	0.8838	0.5961	0.5563	0.069*	
C16	0.7151 (5)	0.5082 (4)	0.4498 (3)	0.0591 (11)	
H16	0.7166	0.4216	0.4745	0.071*	
C17	0.2670 (4)	0.5351 (4)	−0.0126 (3)	0.0493 (9)	
C18	0.1988 (6)	0.5521 (4)	−0.1128 (3)	0.0772 (14)	
H18	0.1445	0.4782	−0.1505	0.093*	
C19	0.2106 (6)	0.6756 (5)	−0.1563 (3)	0.0775 (14)	
H19	0.1640	0.6838	−0.2233	0.093*	
C20	0.2889 (4)	0.7875 (4)	−0.1044 (3)	0.0535 (10)	
C21	0.3603 (5)	0.7708 (4)	−0.0057 (3)	0.0634 (11)	
H21	0.4165	0.8445	0.0312	0.076*	
C22	0.3487 (5)	0.6468 (4)	0.0379 (3)	0.0625 (11)	
H22	0.3982	0.6384	0.1040	0.075*	
C23	1.0176 (5)	0.8341 (4)	0.5968 (3)	0.0619 (11)	
H23A	0.9580	0.7953	0.6508	0.093*	
H23B	1.0731	0.9197	0.6208	0.093*	
H23C	1.0944	0.7750	0.5781	0.093*	
C24	0.2999 (5)	0.9248 (4)	−0.1523 (3)	0.0717 (12)	
H24A	0.2827	0.9139	−0.2264	0.108*	
H24B	0.4045	0.9718	−0.1337	0.108*	
H24C	0.2193	0.9756	−0.1273	0.108*	

### Atomic displacement parameters (Å^2^)

**Table:**

	*U*^11^	*U*^22^	*U*^33^	*U*^12^	*U*^13^	*U*^23^
S1	0.0607 (7)	0.0545 (6)	0.0497 (6)	0.0002 (5)	−0.0136 (5)	0.0075 (5)
O1	0.0739 (19)	0.0560 (17)	0.0772 (19)	0.0045 (14)	−0.0340 (16)	0.0122 (14)
O2	0.0565 (17)	0.0483 (16)	0.0715 (18)	−0.0001 (13)	−0.0165 (14)	0.0049 (13)
N1	0.0499 (18)	0.0470 (18)	0.0431 (17)	0.0026 (14)	−0.0026 (14)	0.0093 (14)
N2	0.057 (2)	0.0468 (19)	0.0492 (18)	0.0056 (15)	−0.0159 (15)	0.0039 (14)
N3	0.056 (2)	0.0458 (18)	0.0542 (19)	0.0054 (15)	−0.0008 (15)	0.0083 (15)
C1	0.063 (3)	0.053 (2)	0.045 (2)	0.0000 (19)	−0.0124 (19)	0.0076 (18)
C2	0.045 (2)	0.047 (2)	0.040 (2)	0.0038 (17)	−0.0019 (16)	0.0018 (16)
C3	0.040 (2)	0.049 (2)	0.041 (2)	0.0107 (17)	−0.0005 (16)	0.0045 (16)
C4	0.058 (2)	0.054 (2)	0.044 (2)	0.0104 (19)	0.0002 (18)	0.0074 (17)
C5	0.071 (3)	0.051 (2)	0.048 (2)	0.006 (2)	0.005 (2)	0.0053 (18)
C6	0.053 (2)	0.053 (2)	0.059 (2)	0.0027 (19)	−0.0004 (19)	0.0033 (19)
C7	0.047 (2)	0.052 (2)	0.044 (2)	0.0109 (18)	0.0027 (17)	0.0064 (17)
C8	0.041 (2)	0.044 (2)	0.0399 (19)	0.0080 (16)	0.0003 (16)	0.0031 (15)
C9	0.049 (2)	0.049 (2)	0.044 (2)	0.0047 (18)	−0.0066 (17)	0.0058 (17)
C10	0.073 (3)	0.052 (3)	0.073 (3)	−0.001 (2)	0.004 (2)	0.007 (2)
C11	0.047 (2)	0.043 (2)	0.047 (2)	0.0043 (17)	−0.0055 (17)	0.0004 (17)
C12	0.062 (3)	0.050 (2)	0.062 (2)	0.004 (2)	−0.023 (2)	0.0092 (19)
C13	0.060 (3)	0.044 (2)	0.074 (3)	0.0064 (19)	−0.015 (2)	0.017 (2)
C14	0.046 (2)	0.041 (2)	0.052 (2)	0.0048 (17)	0.0009 (18)	0.0040 (17)
C15	0.068 (3)	0.049 (2)	0.051 (2)	0.003 (2)	−0.018 (2)	0.0069 (18)
C16	0.074 (3)	0.047 (2)	0.053 (2)	0.005 (2)	−0.018 (2)	0.0157 (18)
C17	0.050 (2)	0.056 (2)	0.040 (2)	0.0031 (19)	−0.0073 (17)	0.0060 (18)
C18	0.104 (4)	0.064 (3)	0.053 (3)	−0.011 (3)	−0.030 (2)	0.012 (2)
C19	0.096 (4)	0.073 (3)	0.056 (3)	−0.002 (3)	−0.028 (2)	0.016 (2)
C20	0.049 (2)	0.061 (3)	0.053 (2)	0.012 (2)	0.0074 (19)	0.014 (2)
C21	0.071 (3)	0.055 (3)	0.059 (3)	−0.008 (2)	−0.006 (2)	0.002 (2)
C22	0.074 (3)	0.067 (3)	0.043 (2)	0.002 (2)	−0.011 (2)	0.012 (2)
C23	0.060 (3)	0.066 (3)	0.054 (2)	0.001 (2)	−0.015 (2)	−0.001 (2)
C24	0.076 (3)	0.068 (3)	0.072 (3)	0.010 (2)	0.009 (2)	0.018 (2)

### Geometric parameters (Å, °)

**Table:**

S1—C7	1.721 (3)	C10—H10B	0.9600
S1—C2	1.749 (4)	C10—H10C	0.9600
O1—C9	1.225 (4)	C11—C12	1.380 (5)
O2—C14	1.363 (4)	C11—C16	1.392 (5)
O2—C23	1.427 (4)	C12—C13	1.363 (5)
N1—C1	1.282 (4)	C12—H12	0.9300
N1—C2	1.391 (4)	C13—C14	1.391 (5)
N2—C9	1.354 (4)	C13—H13	0.9300
N2—C11	1.407 (4)	C14—C15	1.366 (5)
N2—H2	0.8600	C15—C16	1.381 (5)
N3—C10	1.452 (4)	C15—H15	0.9300
N3—C6	1.463 (4)	C16—H16	0.9300
N3—C5	1.469 (5)	C17—C22	1.367 (5)
C1—C17	1.450 (5)	C17—C18	1.397 (5)
C1—H1	0.9300	C18—C19	1.366 (5)
C2—C3	1.387 (4)	C18—H18	0.9300
C3—C8	1.440 (5)	C19—C20	1.366 (6)
C3—C9	1.485 (5)	C19—H19	0.9300
C4—C5	1.516 (5)	C20—C21	1.387 (5)
C4—C8	1.506 (4)	C20—C24	1.516 (5)
C4—H4A	0.9700	C21—C22	1.371 (5)
C4—H4B	0.9700	C21—H21	0.9300
C5—H5A	0.9700	C22—H22	0.9300
C5—H5B	0.9700	C23—H23A	0.9600
C6—C7	1.502 (5)	C23—H23B	0.9600
C6—H6A	0.9700	C23—H23C	0.9600
C6—H6B	0.9700	C24—H24A	0.9600
C7—C8	1.360 (5)	C24—H24B	0.9600
C10—H10A	0.9600	C24—H24C	0.9600
			
C7—S1—C2	91.41 (17)	H10B—C10—H10C	109.5
C14—O2—C23	117.7 (3)	C12—C11—C16	117.2 (3)
C1—N1—C2	121.1 (3)	C12—C11—N2	118.4 (3)
C9—N2—C11	128.4 (3)	C16—C11—N2	124.4 (3)
C9—N2—H2	115.8	C13—C12—C11	121.9 (3)
C11—N2—H2	115.8	C13—C12—H12	119.0
C10—N3—C6	110.1 (3)	C11—C12—H12	119.0
C10—N3—C5	110.9 (3)	C12—C13—C14	120.7 (3)
C6—N3—C5	109.3 (3)	C12—C13—H13	119.6
N1—C1—C17	123.4 (3)	C14—C13—H13	119.6
N1—C1—H1	118.3	O2—C14—C15	126.2 (3)
C17—C1—H1	118.3	O2—C14—C13	115.9 (3)
C3—C2—N1	125.1 (3)	C15—C14—C13	117.9 (3)
C3—C2—S1	111.3 (3)	C14—C15—C16	121.4 (3)
N1—C2—S1	123.6 (2)	C14—C15—H15	119.3
C2—C3—C8	111.7 (3)	C16—C15—H15	119.3
C2—C3—C9	126.5 (3)	C15—C16—C11	120.7 (3)
C8—C3—C9	121.8 (3)	C15—C16—H16	119.6
C5—C4—C8	111.0 (3)	C11—C16—H16	119.6
C5—C4—H4A	109.4	C22—C17—C18	116.7 (3)
C8—C4—H4A	109.4	C22—C17—C1	123.5 (3)
C5—C4—H4B	109.4	C18—C17—C1	119.7 (3)
C8—C4—H4B	109.4	C19—C18—C17	121.0 (4)
H4A—C4—H4B	108.0	C19—C18—H18	119.5
N3—C5—C4	112.0 (3)	C17—C18—H18	119.5
N3—C5—H5A	109.2	C18—C19—C20	121.9 (4)
C4—C5—H5A	109.2	C18—C19—H19	119.0
N3—C5—H5B	109.2	C20—C19—H19	119.0
C4—C5—H5B	109.2	C19—C20—C21	117.4 (4)
H5A—C5—H5B	107.9	C19—C20—C24	121.8 (4)
N3—C6—C7	109.9 (3)	C21—C20—C24	120.8 (4)
N3—C6—H6A	109.7	C22—C21—C20	120.7 (4)
C7—C6—H6A	109.7	C22—C21—H21	119.7
N3—C6—H6B	109.7	C20—C21—H21	119.7
C7—C6—H6B	109.7	C21—C22—C17	122.2 (3)
H6A—C6—H6B	108.2	C21—C22—H22	118.9
C8—C7—C6	124.7 (3)	C17—C22—H22	118.9
C8—C7—S1	112.6 (3)	O2—C23—H23A	109.5
C6—C7—S1	122.6 (3)	O2—C23—H23B	109.5
C7—C8—C3	113.0 (3)	H23A—C23—H23B	109.5
C7—C8—C4	119.7 (3)	O2—C23—H23C	109.5
C3—C8—C4	127.4 (3)	H23A—C23—H23C	109.5
O1—C9—N2	123.0 (3)	H23B—C23—H23C	109.5
O1—C9—C3	120.2 (3)	C20—C24—H24A	109.5
N2—C9—C3	116.9 (3)	C20—C24—H24B	109.5
N3—C10—H10A	109.5	H24A—C24—H24B	109.5
N3—C10—H10B	109.5	C20—C24—H24C	109.5
H10A—C10—H10B	109.5	H24A—C24—H24C	109.5
N3—C10—H10C	109.5	H24B—C24—H24C	109.5
H10A—C10—H10C	109.5		
			
C2—N1—C1—C17	179.1 (3)	C2—C3—C9—O1	−162.9 (4)
C1—N1—C2—C3	168.5 (4)	C8—C3—C9—O1	14.4 (5)
C1—N1—C2—S1	−10.3 (5)	C2—C3—C9—N2	16.0 (5)
C7—S1—C2—C3	0.7 (3)	C8—C3—C9—N2	−166.7 (3)
C7—S1—C2—N1	179.6 (3)	C9—N2—C11—C12	173.1 (4)
N1—C2—C3—C8	−179.6 (3)	C9—N2—C11—C16	−8.3 (6)
S1—C2—C3—C8	−0.7 (4)	C16—C11—C12—C13	0.4 (6)
N1—C2—C3—C9	−2.1 (6)	N2—C11—C12—C13	179.1 (4)
S1—C2—C3—C9	176.8 (3)	C11—C12—C13—C14	−1.0 (7)
C10—N3—C5—C4	170.6 (3)	C23—O2—C14—C15	0.0 (5)
C6—N3—C5—C4	−67.8 (4)	C23—O2—C14—C13	−179.4 (3)
C8—C4—C5—N3	45.9 (4)	C12—C13—C14—O2	−179.4 (4)
C10—N3—C6—C7	173.8 (3)	C12—C13—C14—C15	1.1 (6)
C5—N3—C6—C7	51.6 (4)	O2—C14—C15—C16	179.8 (4)
N3—C6—C7—C8	−20.0 (5)	C13—C14—C15—C16	−0.8 (6)
N3—C6—C7—S1	161.5 (3)	C14—C15—C16—C11	0.3 (7)
C2—S1—C7—C8	−0.5 (3)	C12—C11—C16—C15	−0.1 (6)
C2—S1—C7—C6	178.1 (3)	N2—C11—C16—C15	−178.7 (4)
C6—C7—C8—C3	−178.4 (3)	N1—C1—C17—C22	1.5 (6)
S1—C7—C8—C3	0.1 (4)	N1—C1—C17—C18	−179.2 (4)
C6—C7—C8—C4	0.5 (5)	C22—C17—C18—C19	−1.6 (7)
S1—C7—C8—C4	179.1 (3)	C1—C17—C18—C19	179.0 (4)
C2—C3—C8—C7	0.4 (4)	C17—C18—C19—C20	0.0 (8)
C9—C3—C8—C7	−177.3 (3)	C18—C19—C20—C21	1.5 (7)
C2—C3—C8—C4	−178.4 (3)	C18—C19—C20—C24	−179.1 (4)
C9—C3—C8—C4	3.9 (6)	C19—C20—C21—C22	−1.4 (6)
C5—C4—C8—C7	−12.8 (5)	C24—C20—C21—C22	179.3 (4)
C5—C4—C8—C3	165.9 (3)	C20—C21—C22—C17	−0.3 (7)
C11—N2—C9—O1	−0.3 (6)	C18—C17—C22—C21	1.8 (6)
C11—N2—C9—C3	−179.2 (3)	C1—C17—C22—C21	−178.9 (4)

### Hydrogen-bond geometry (Å, °)

**Table:**

*D*—H···*A*	*D*—H	H···*A*	*D*···*A*	*D*—H···*A*
N2—H2···N1	0.86	2.12	2.807 (4)	137
C1—H1···S1	0.93	2.60	3.051 (4)	110
C16—H16···O1	0.93	2.27	2.863 (5)	121
